# Case of Chronic Ocular Hyperœsthesia

**Published:** 1856-04

**Authors:** Sanford B. Hunt


### ART. II.—*Case of Chronic Ocular Hyperæsthesia.*

By Sanford B. Hunt, M. D.

The ophthalmic surgeon, more perhaps than any other specialist, requires an intimate familiarity with the phenomena of reflex action especially, as well as the pathology and therapeutics of those numerous disorders of the general system which reäct upon the organ he assumes to treat. In all the various departments of surgical science which have been more commonly assigned to specialists, none so much requires the physician and pathologist as ophthalmic surgery. Yet unthinking common consent has named this as the most appropriate field for specialism.

The records of ophthalmic science, however, prove to us, that it is *not* specially indebted to *oculists*, for its rapid and triumphant progress. Men of general practice, devoting themselves to the human system as an inseparable unit, are they who have accurately defined ophthalmic pathology, devised new methods of treatment, discovered new facts in the anatomy of the organ, and invented new operations for the relief of its diseases and deformities. How few are the names of oculists which have any permanent record? and those few, how feebly do they compare with those of Lawrence, Mackenzie, Mayo and Jager!

Cases of ocular hyperæsthesia are not common. Mackenzie describes its symptoms as follows: "The chief characteristic of the affection is great intolerance of light, with which are combined, in a greater or less degree, photopsia, chrupsia, pain in the eye and head, augmented tactual sensibility of the eyeball and eyelids, and spasmodic contraction of the orbicularis palpebrarum. So far as it implicates the fifth nerve and portio dura of the seventh, it appears to be the effect of a reflex action communicated to them, in consequence of the excited state of the retina and optic nerve."

This latter statement, I take it, should only be understood as applying to the acute variety, or to those very rare cases of the chronic form depending upon immediate injury to the retina. For Mackenzie himself states, a little farther on, that the chronic form depends usually on some constitutional disturbance. Again, it is equally evident that the acute form may occur entirely independent of any excitation of the retina. The only symptoms mentioned by Mackenzie which necessarily imply disturbance of the retina, are photopsia and chrupsia, and both of these are not unfrequently wanting.

Reserving any further remarks upon the essential nature of the disease, I shall here introduce an illustrative case of the chronic variety, which has lately been, and still is, under my care.

Miss G., a resident of Buffalo, has suffered for some months from intolerance of light, so great as to necessitate her seclusion from society, and confinement in a darkened room. She suffered also from severe circumorbital pain.

About the first of September, 1855, a physician was called, whose diagnosis was understood to be retinitis, threatening amaurosis. I am unable to describe the treatment, further than that strychnia was given internally, with a marked increase of the unpleasant symptoms following its administration. No improvement occurring, the physician in charge discontinued his attendance, and after remaining some time without treatment, I was called on the 28th of Dec., to assume the case.

The intolerance of light is not excessive, thought sufficient to confine her to a darkened room. Twilight is more agreeable than darkness; in fact, if the room is very dark, pain is produced by the effect at vision. Vision itself is clear and distinct, the outlines of objects being well defined, and her estimate of the relief or depression of any object accurate.

These facts alone were sufficient to form a negative diagnosis as to retinitis. In *pure* retinitis no pain occurs, neither is intolerance of light a necessary accompaniment, inasmuch as, like pain, it depends upon the implication of the choroid coat in the inflammatory action. Again, a retinitis of this duration, however moderate, must have ere this produced at least a partial amaurosis. Again, had there been even mere excitation of the retina, photopsia, or chrupsia, should have been presented. On the contrary, these symptoms were wanting, and vision was as acute as natural. The fact of positive darkness being painful, seemed to indicate that the dilatation of the pupil was the cause.

The pain, as described, was circumorbital, paroxysmal, and imperfectly periodic. The increase of tactual sensibility was tolerably well marked. Blepharospasm was present, but by no means so prominently as in scrofulous ophthalmia. Any fatigue of the eye produced it, and very much augmented the pain.

The aspect of the eye was natural, except that the pupil was closely contracted, in a room only sufficiently lighted for an examination. From the point of insertion of each of the recti muscles, two or three dilated vessels ran toward the cornea as in rheumatism of those muscles.

Nothing in the history of the patient indicated that the eye had been subjected to intense light or great fatigue.

Upon inquiry, I learned that facial neuralgia accompanied the ophthalmic symptoms, involving nearly all the branches of the fifth pair. Her health was impaired; the appetite defective; muscular tone and energy deficient; some degree of spinal irritation was present; and she had also slight leucorrhœa, with distinct and occasionally severe ovarian irritation.

From this train of symptoms it was evident that the general health alone was at fault, and that the hyperæsthesia might well be considered as a neuralgia of the fifth pair, involving particularly the ciliary nerves.

The treatment was simple. No local means were used, with the exception of a liniment of chloroform to the parts supplied by the supra-orbital and infra-orbital nerves. Its formula was the following:

℞ Chloroform,
Gum camphor,
Olive oil, āā equal parts.

The immediate effect of this liniment was to entirely suspend the hyperæsthesia for a time, giving a brief but perfect relief to all the symptoms; thus confirming the neuralgic diagnosis.

The general treatment was directed to the restoration of the powers of the system, and to the alleviation of pain.

As a tonic I prescribed:

℞ Ferri et quiniæ citratis, ʒj.

Vini Oporto, ℥ij. Misce.

Dose, a teaspoonful three times daily.

As a nervous anodyne, the following:

℞ Zinci Valerianatis, grs. xx.
Gum tragacanthis, grs. xx.
Aqua, q. s.
Div. in pilulis xx.

Take one at bedtime.

Under the persevering use of these remedies, with as much exercise in the way of riding or walking as the condition of the eyes would admit, the eyes and general health improved pari passu. She soon became able to bear the light to which the other members of the family were accustomed, and shortly after was able to read for a few minutes at a time. During the early part of February she overtasked her eyes by spending an entire day in reading, but recovered from the effects of it in a week or two. She cannot yet be considered as recovered, but is fairly convalescent.

This is, probably, a fair instance of ocular hyperæsthesia as it occurs in the chronic form, depending upon constitutional derangements. Dr. Isaac Hays reported, many years since, two or three cases of a far more acute type, in which, though it was strictly a reflex action manifesting itself through the ocular branches of the fifth pair, the point of irritation was found in the teeth, and the disease was entirely removed by the extraction of decayed teeth. (In one instance, I believe, there was an exostosis of the root.)

Of the causes of ocular hyperæsthesia, it is evident that there must be many, especially the chronic variety. Any irritation which may be reflected upon, and expressed through the fifth pair, is capable of producing it, and as the fifth pair is especially the nerve of emotion, and is, in fact, the only one strictly devoted to its expression, it is evident that its sympathies extend far beyond its own distribution. The strong light and heat incident to an exposure to a July sun have caused it. In the well-known case of Mr. Goadby, the microscopist, it was occasioned by a sudden flash of brilliant sunlight through a microscope.

The period of continuance of the disease is said to be uncertain. Not unfrequently it lasts many months, but the almost uniform termination is in recovery. Sometimes the photophobia is so intense, and the sensibility of the surrounding parts so great, as almost to preclude an examination. It is probable that in these cases the use of chloroform, locally, might obviate the difficulty.

The presence or absence of photopsia or chrupsia, will furnish a pretty sure index of the implication or the retina or choroid, while the absence of spasm of the eyelids will indicate that the seventh pair does not respond to the stimulus to the fifth.

March 6th, 1856.

